# Is Treatment-Emergent Toxicity a Biomarker of Efficacy of Apatinib in Gastric Cancer?

**DOI:** 10.1200/JCO.2016.68.8663

**Published:** 2016-08-15

**Authors:** Hyo Jin Lee, Ji Young Moon, Seung Woo Baek

**Affiliations:** Hyo Jin Lee, Chungnam National University and Chungnam National University Hospital, Daejeon, Republic of Korea; Ji Young Moon and Seung Woo Baek, Chungnam National University Hospital, Daejeon, Republic of Korea

## To the Editor:

Li et al^1^ reported the results of a randomized, double-blind, placebo-controlled phase III trial of apatinib, which showed significant survival benefits in patients with chemotherapy-refractory advanced or metastatic adenocarcinoma of the stomach or gastroesophageal junction. These results validate the role of vascular endothelial growth factor receptor (VEGFR) -2 signaling as an important therapeutic target; however, the clinical effects of apatinib are modest, with limited survival prolongation compared with placebo—median overall survival, 6.5 months versus 4.7 months; median progression-free survival, 2.6 months versus 1.8 months, respectively—and a low objective response rate of 1.70% as assessed by an independent response evaluation committee.^1^ Therefore, it is critically challenging to identify suitable predictive biomarkers that could be used to select patients who will benefit most from VEGFR-2 signal-inhibiting agents, such as apatinib, which would thereby improve efficacy and avoid unnecessary toxicity and high cost. These biomarkers might come from the cellular or molecular level using biospecimens collected from patients. Alternatively, occurrence of adverse events might act as surrogate biomarkers of drug activity, enabling the prediction of outcome during treatment because the occurrence of treatment-emergent toxic effects is associated with a pharmacodynamic effect of the drug.^2-4^ Recently, it has been suggested that the occurrence of specific adverse events, such as hypertension, hand-foot syndrome, and proteinuria, during antiangiogenic therapy might be associated with improved efficacy.^4-7^ Regarding apatinib, in particular, it was reported that hypertension and hand-foot skin reaction were significantly related to longer progression-free and overall survival in patients with advanced breast cancer.^8^ Therefore, it would be interesting to know whether the prospective data set reported by Li et al^1^ shows that the development of treatment-specific adverse effects, such as hypertension, hand-foot syndrome, and proteinuria, is related to treatment outcome.

The investigators could help to address this issue by analyzing survival data according to the emergence of treatment-related adverse events. Such data could help clinicians make better treatment decisions and may shed light on the future development of VEGFR signaling-targeted therapy for gastric and gastroesophageal junction carcinomas.
